# Adherence, Fears, and Beliefs about Biologic Drugs in Rheumatoid Arthritis Patients: A North African Pilot Study

**DOI:** 10.31138/mjr.200823.afa

**Published:** 2024-12-31

**Authors:** Soumaya Boussaid, Emna Hannech, Sonia Rekik, Safa Rahmouni, Khaoula Zouaoui, Maissa Abbes, Hela Sahli, Mohamed Elleuch, Helmi Ben Saad

**Affiliations:** 1 Rheumatology Department, La Rabta Hospital, Tunis, Tunisia,; 2 Faculty of Medicine of Tunis, University Tunis el Manar, Tunis, Tunisia,; 3 Reaserch Unit LR 05 SP 01, La Rabta Hospital, Tunis, Tunisia,; 4 University of Sousse, Faculty of Medicine of Sousse, Farhat Hached Hospital, Sousse, Research Laboratory “Heart Failure, LR12SP09”, Sousse, Tunisia

**Keywords:** beliefs, biologic drugs, fears, medication adherence, rheumatoid arthritis

## Abstract

**Purpose:**

To investigate the impact of beliefs in adherence to biologic drugs among patients with rheumatoid arthritis (RA).

**Methods:**

This was a cross-sectional study, including RA patients who were on biologic disease-modifying antirheumatic drugs (bDMARDs). Therapeutic adherence was evaluated arbitrarily using a self-reported method by asking them the following question: “Do you regul arly take your biologic drug as prescribed by your doctor?”. The Beliefs about Medicines Questionnaire (BMQ) was used to evaluate medication beliefs [general overuse, general harm, specific necessity, specific concerns].

**Results:**

Seventy-five RA patients were included (80.0% females, 33.3% illiterate, and 68.0% unemployed, mean age: 57±9 years, mean disease activity score: 3.94±1.32). Adherence to the current biologic drug was reported by 71 patients (94.7%). The means ± standard deviation scores for general overuse, general harm, specific necessity, and specific concerns were 14.0±2.4, 10.8±4.4, 20.6±5.7, and 10.3±3.3, respectively. Compared to the adherent group (n=71), the non-adherent group (n=4) had a lower specific necessity score (21.0±5.4 vs. 13.5±7.5, respectively, p=0.009), a higher specific concern score (10.1±3.13 vs. 15.0±2.8, respectively, p=0.036), and similar scores for general overuse and general harm (10.0±2.3 vs. 9.5±1.9, p=0600; 13.3±4.4 vs. 11.5±4.1, p=0.400, respectively). In logistic regression, specific necessity and specific concerns scores were significantly associated with adherence (Odds-ratio (OR)= 0.855, 95% confidence interval (CI) [0.726–1.006], and 1.438, 95% CI [1.004–1.980], respectively).

**Conclusion:**

Our study showed that RA patients have strong beliefs about the necessity to take biologic drugs which significantly influence the adherent behaviour therapy.

## KEY POINTS

- Belief about the necessity of biologic drugs are important for RA patients’.
- Beliefs about biologic drugs have been identified as key contributors to adherence.

## INTRODUCTION

Rheumatoid arthritis (RA) is a chronic autoimmune disorder characterised by joint inflammation accompanied by pain, swelling, damage, and disability.^1^ RA affects significantly people's quality of life (QoL).^2^ Ongoing monitoring on the use of chronic therapies are therefore required.^1^ Currently, various therapies are offered with new strategies.^3^ Biologic disease-modifying anti-rheumatic drugs (bDMARDs) are therapies targeting pro-inflammatory cytokines or cellular receptors involved in the inflammatory process of chronic rheumatic diseases.^4^ bDMARDs, which are prescribed for a long period of time, require appropriate adherence behavior.^5^

Adherence to disease-modifying anti-rheumatic drugs (DMARDs) is suboptimal, ranging from 22% (underuse) to 107% (overuse).^6^ More specifically, adherence to bDMARDs is highly variable, ranging from 30 to 89%.^7–9^ Adherence to bDMARDs can be influenced by a number of factors linked to the patient and to the disease.^10^ Patients’ adherence to bDMARDs is critical to enhance their compliance.^11^ RA patients develop a system of fear and beliefs about their illness and therapy.^5^ Indeed, past personal experiences, experiences of others, and the impact of the illness feed their prejudices about treatment.^12,13^ These beliefs may affect pain and disease outcomes as well as treatment tolerance and acceptance.^14^ Patients’ beliefs, therefore, affect patients’ QoL, thus, possibly having a high economic burden for the healthcare system.^15^ In previous studies, beliefs about medicine have been demonstrated to be a determining factor in patients' adherence to the treatment of chronic diseases.^16^ Having an overview of patients’ medical perceptions and beliefs is therefore crucial in order to prevent treatment inefficiency and reduce non-adherence.^6^ However, this topic is poorly documented in the literature, especially studies focusing on biologic drug adherence and beliefs in RA patients.

Therefore, the purposes of this study were to identify the beliefs of RA patients about biologic drugs, and to assess whether these beliefs are predictors of non-adherence behavior.

## PATIENTS AND METHODS

### Study design

This was a cross-sectional study performed from August 2020 to June 2021 at two rheumatology departments in two university hospital institutions (ie; La Rabta Hospital, and the Military Hospital, Tunis, Tunisia). The study was approved by La Rabta Hospital ethics committee (approval number: RH1904/2020). Written informed consent to participate in the study was obtained from all patients.

The study was performed during the pandemic of coronavirus disease (COVID-19). All recommended preventive measures to fight against the COVID-19 transmission (eg; physical distance of at least one meter from others, wearing a fitted face mask, properly, cleaning hands frequently with alcohol-based hand rub or soap, and water freezing friction) were applied.

### Population

**Figure 1.**
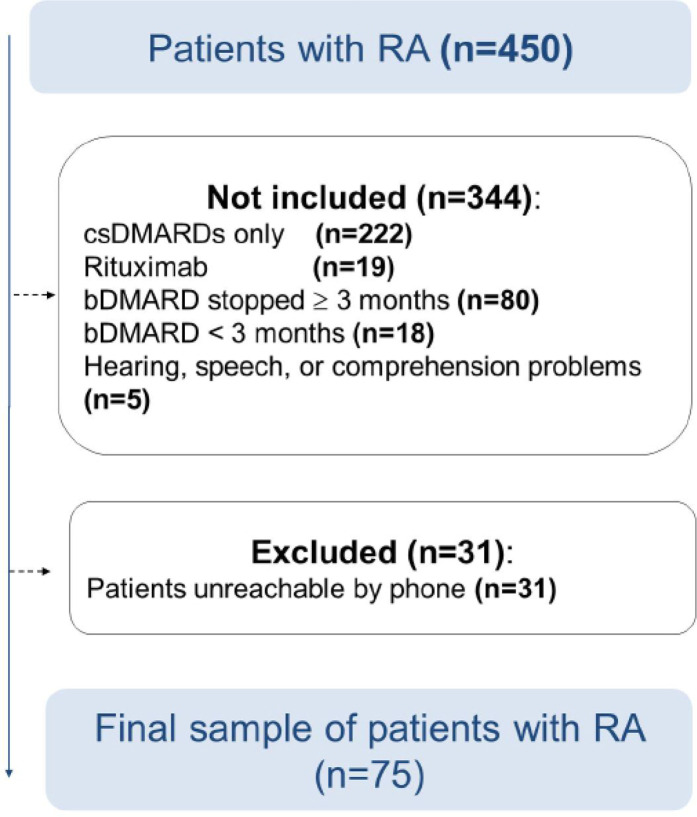
Final patient analysis.

The population source was the RA patients meeting the American College of Rheumatology/European League Against Rheumatism (ACR/EULAR) 2010 criteria.^17^ The population target was RA patients consulting the aforementioned two rheumatology departments during the study period. Only RA patients aged > 18 years and treated with bDMARDs, associated or not with conventional synthetic Disease-Modifying Anti-Rheumatic Drugs (csDMARDs), for at least three months were included in the study. Only patients on tumor necrosis factor inhibitor (TNFi) or interleukin-6 receptor inhibitor (IL-6 Ri) drugs were eligible. Patients who were unable to understand and answer the different questionnaire items because of hearing, speech, or comprehension problems were not included in the study. Patients unreachable by phone after two calls at one week intervals were excluded from the final analysis (**Figure 1**).

### Sample size

The sample size was calculated according to the following predictive equation : N = (Z_α/2_^2^ p q)/ê^2^; where “n” was the number of needed RA patients; “Z_α/2_” was the normal deviate for type 1 error (Z_α/2_ = 1.96 for 5% level of significance); “q” was equal to “1-p”; “Δ” was the accuracy (=7%), and “p” was the frequency of the main outcome (ie; adherence of RA patients to bDMARD).^18^ According to Mean-Vazquez et al. 88.8% (p=0.888) of RA patients showed good adherence to bDMARDs. The sample size was therefore 78 consecutive RA patients.^9^ The assumption of 83% of non-inclusion and exclusion criteria gave a revised sample of 460 RA patients (460 = 78/(1−0.83)).

### Data collection and investigated variables

Patients were interviewed during their hospital stay or during their medical visit. A medical questionnaire including two parts was applied.

The first part, which took about 10 minutes, was reserved to collect the following data: sex, age, schooling level (ie; illiteracy, primary school, secondary school, university), profession (ie; unemployed, employed, retired), marital status (ie; celibate, married, divorced, widowed), and living area (ie; urban, rural). The following disease characteristics were noted from the patients’ medical record sheet: disease duration (years), immunologic profile [eg; positive rheumatoid factor (RF), positive anti-citrullinated protein antibodies (ACPA)], presence (and location) of extra-articular manifestations, presence of coxitis and/or atlanto-axial dislocation, number of nocturnal awakenings, duration of morning stiffness (minutes), tender and swollen joints counts, patient global evaluation, pain (visual analogue scale (VAS)), functional impairment with the health assessment questionnaire (HAQ), C-reactive protein (CRP, mg/l), disease activity score 28-CRP, bDMARD (eg; type, duration, rank, administration route), and uptake of csDMARD.^19,20^ VAS pain is a 100 mm line, with two end points: 0 ‘no pain' and 100 'extreme pain'.^21^ The HAQ is a self-reported and valid questionnaire for assessing functional disability in patients with RA. It was translated and validated in Arabic language.^22^ A CRP value > 6 mg/l was considered abnormal.^23^

The second part of the questionnaire concerns the assessment of medicine beliefs. Patients' beliefs about their biological treatment were evaluated via the French validated version of the beliefs about medicine (BMQ) questionnaire.^24^ The BMQ, which was developed based on the literature and patients’ interviews, is validated in multiple languages in several chronic disease patients.^25,26^ The BMQ has been used to assess the therapeutic beliefs of patients followed for chronic rheumatic diseases, such as RA and spondyloarthritis under conventional treatment and/or biologics.^27–30^ The BMQ assesses the perceived need to remain in the treatment, the perceived effectiveness of the treatment, and concerns about side effects.^24^ The BMQ consists of 18 components divided into two areas: i) General beliefs: four elements for general overuse and four elements for general harm; and ii) Specific beliefs: five elements for specific necessity and five elements for specific concern with respect to the current biologic drugs. The general overuse and harm scores vary between 4 and 20. Higher general overuse scores indicate a more negative opinion about physicians’ attitudes and prescriptions. Higher general harm scores indicate a more negative opinion regarding the drugs’ adverse effects. The scales of specific necessity and concerns vary between 5 and 25. Specific necessity scores near the top end represent stronger perceptions of personal need for biologic drugs. Specific concern scores near the upper limit indicate more significant concerns about the adverse effects of biologic drugs. Patients answered each item using a 5-point Likert scale (1 = strongly disagree, 5 = strongly agree). The French version of the BMQ was translated into the Tunisian dialect. The latter was not validated. However, before its application to patients, the BMQ was discussed between the two physicians who conducted the survey (SB and EH in the authors’ list) and one leader of the study (ME in the authors’ list). The objectives of the discussion were to clarify certain questions and to standardise the way of asking them. In addition, a “role play” was carried out and several scenarios for answering the questions were discussed. The BMQ was completed by the patients if they can read and understand, and in case of analpahebtic patients, a referral physician (SB or EH in the authors’ list) interviewed the patients face to face. The BMQ took about 10 minutes.

### Assessment of adherence to current biologic drugs

Up to July 2022, there is no gold standard for measuring adherence to biologic drugs.^6,31^ In this study, adherence to treatment was evaluated arbitrarily using a self-reported method by asking patients the following question: “Do you regularly take your biologic drug as prescribed by your doctor?”. Patients who answered ‘yes’ were considered ‘adherents’ to treatment, and those who answered ‘no’ were considered ‘non-adherents’ to treatment.

### Statistical analysis

#### Data expression

The descriptive study included the calculation of absolute and relative frequencies for categorical variables, and means±standard deviations (minimum and maximum) for quantitative variables.

#### Comparison between groups

Student t-test and 2-sided chi-2 test were used to compare quantitative and categorical data of two independent groups (eg; adherent vs. non-adherent). One-way analysis of variance (ANOVA), and, if applicable, Tukey’s post hoc test were used to compare quantitative data of three groups or more (e.g., schooling level, profession, and marital status).

#### Correlation analysis

The Pearson correlation coefficient “r” between the specific necessity score and some outcomes (eg; patient global evaluation; VAS pain, number of nocturnal awakenings, tender joint count, swollen joint count, health assessment questionnaire, disease activity score 28-CRP) was calculated. The Pearson “r” was considered “high” when it was > 0.70, “good” when it was between 0.50–0.70, “fair” if it was between 0.30–0.50 and “weak or no association” if it was < 0.30.^32^

#### Multivariate analysis

In multivariate analysis, the search for risk factors was performed by calculating the odds ratio (OR) with a 95% confidence interval (CI).

The study data were entered and analysed using Statistical Package for Social Sciences (SPSS) version 23.0 software. Throughout the statistical study, the significance level (p) was set at 0.05.

## RESULTS

Among the 450 RA patients included in the initial step, only 75 were enrolled in the final analysis (**Figure 1**).

### Descriptive data

**Table 1** details the patients’ characteristics and disease features. The general profile of patients was dominated by the female sex (80%), illiteracy and primary school levels (70.6%), unemployed patients (68.0%), married status (85.3%), and the urban area (88.0%). The most frequent extra-articular manifestations were pulmonary and rheumatoid nodules (14.7% each), and the most used three bDMARDs were Infliximab, Certolizumab, and Tocilizumab (22.7% each). The most frequent bDMARDs route of administration was the subcutaneous one (54.7%). Sixty-four percent of patients were treated by csDMARD.

**Table 1.** Patients’ characteristics and disease features (n=75).

**Parameters**	**Unit/category**		**Numerical data**
**Sex**	Female		60 (80.0)
**Age**	Years		57±9 [34–80]
**Schooling level**	Illiteracy		25 (33.3)
	Primary school		28 (37.3)
	Secondary school		19 (25.3)
	University		3 (4.0)
**Profession**	Unemployed		51 (68.0)
	Employee		20 (26.7)
	Retired		4 (5.3)
**Marital status**	Celibate		5 (6.7)
	Married		64 (85.3)
	Divorced/widowed		6 (8.0)
**Living area**	Urban		66 (88.0)
	Rural		9 (12.0)
**Disease duration**	Years		15±80 [2–39]
**Immunologic profile**	Positive rheumatoid factor		44 (58.7)
	Positive anti-citrullinated protein antibodies		40 (53.3)
**Extra-articular manifestations**	Pulmonary		11 (14.7)
	Rheumatoid nodules		11 (14.7)
	Ocular		3 (4.0)
	Cardiac		1 (1.3)
	Kidney		2 (2.7)
	Total		27 (36.0)
**Coxitis**	Yes		3 (4.0)
**Atlanto-axial dislocation**	Yes		6 (8.0)
**Number of nocturnal awakenings**			0.97±1.27 [0.00–4.00]
**Duration of morning stiffness**	Minutes		8.10±9.95 [0.00–30.00]
**Tender joints count**			6.12±5.72 [0.00–28.00]
**Swollen joints count**			2.92±3.79 [0.00–18.00]
**Patient global evaluation**			5.57±1.87 [1.00–8.00]
**Visual analogic scale pain**	mm		57.46±20.47 [0.00–90.00]
**C-reactive protein (CRP)**	mg/l		19.38±33.55 [0.00–200.00]
	> 6 mg/l		31 (41.3)
**Disease activity score 28-CRP**			3.94±1.32 [1.21–7.15]
**Biologic disease modifying antirheumatic drugs (bDMARDs)**	Type	Infliximab	17 (22.7)
		Certolizumab	17 (22.7)
		Tocilizumab	17 (22.7)
		Etanercept	16 (21.3)
		Golimumab	1 (1.3)
	Duration	Months	37.17±39.44 [4.00–24.01]
	Rank		1.41±0.9 [1.00–5.00]
	Route administration	Subcutaneous	41 (54.7)
		Intra-venous	34 (45.3)
**Conventional synthetic modifying antirheumatic drugs**	Yes		48 (64.0)

Quantitative data were mean±standard deviation [minimum–maximum]. Categorical data were number (%).

### Disease-related data and bDMARDs adherence

Adherence to current biologic drugs was reported by 71 patients (94.7%). **Table 2** details the relationship between patient characteristics, disease findings, and adherence to the biologic drug. It appears that only the number of nocturnal awakenings influences the adherence rate: compared to the adherent patients, the non-adherent ones had a higher number of nocturnal awakenings (1.00±1.30 vs. 1.10±1.20, respectively, p=0.03) (**Table 2**).

**Table 2.** Relationship between patient characteristics, disease findings, and adherence to the biologic drug (n=75).

**Parameter**	**Unit/category**		**Adherent patient (n=71)**	**Non-adherent patient (n=4)**	p-value*
**Sex**	Female		57 (80.3)	3 (75)	0.50
**Age**	Years		57.12±8.78	53.25±14.45	0.40
	Illiteracy		24 (33.8)	1 (25.0)	0.70
**Schooling level**	Primary school		25 (35.2)	3 (75.0)	0.10
	Secondary school		19 (26.76)	0 (0.0)	0.20
	University		3 (4.2)	0 (0.0)	-
	Unemployed		48 (67.6)	3 (75.0)	0.80
**Profession**	Employee		19 (26.8)	1 (25.0)	0.81
	Retired		4 (5.6)	0 (0)	-
	Celibate		5 (7.0)	1 (25.0)	0.13
**Marital status**	Married		62 (87.3)	2 (50.0)	0.10
	Divorced or widowed		5 (7.0)	1 (25.0)	0.19
**Living area**	Urban area		9 (12.7)	0 (0.0)	-
	Rural area		62 (87.3)	4 (100)	0.50
**Patient global evaluation**	cm		5.50±2.00	5.90±1.80	0.30
**Visual analogue scale**	mm		57.91±20.84	58.61±21.13	0.20
**Number of nocturnal awakenings**			1.00±1.30	1.10±1.20	0.03^*^
**Tender joint count**			8.20±8.80	8.00±9.90	0.10
**Swollen joint count**			6.70±5.90	6.90±6.00	0.70
**Swollen joint count**			3.20±4.10	3.40±3.90	0.60
**Disease activity score 28- C-reactive protein**			4.21±1.16	4.07±1.35	0.40
**Health assessment questionnaire**			0.60±0.40	0.76±0.54	0.50
**Biologic disease modifying anti-rheumatic drugs**	Duration	months	38.20±40.20	17.70±6.30	0.10
	Rank		1.38±0.86	2.00±1.41	0.22
	Route administration	Subcutaneous	39 (54.9)	2 (50.0)	0.84
		Intra-venous	32 (45.1)	2 (50.0)	0.84
**Conventional synthetic disease modifying anti-rheumatic drugs**	Yes		26 (36.6)	1 (25.0)	0.50
	No		45 (63.4)	3 (75.0)	0.50

Quantitative data were mean±standard deviation. Categorical data in number (%).

* p-value (2-sided chi-2 test or student t test) <0.05: comparison between the 2 groups.

### BMQ findings

**Table 3** illustrates the distribution of responses for each item on the BMQ. The main conclusions of this table are:

- Concerning the “general overuse” item, its mean±SD (range) score was 14.0±2.4 [6.0–20.0]. Most patients (82.7%) believed that “doctors overuse the medicines”, 92.0% didn’t believe that “natural remedies are safer than medicines”, 88.0% believed that “doctors place too much trust on medicines”, and 58.7% believed that “if doctors had more time with patients they would prescribe fewer medicines”.
- Concerning the “general harm” item, its mean±SD (range) score was 10.8±4.4 [4.0–20.0]. Most patients (82.7%) believed that “people who take medicines should stop their treatment for a while every now and again”, 72% believed that “most medicines are addictive”, 49.3% didn’t believe that “medicines do more harm than good”, and 48.0% believed that “all medicines are poisons”.
- Concerning the “specific necessity” item, its mean±SD (range) score was 20.6±5.7 [5.0–25.0]. Most patients (77.3%) believed that “their healths depend on biologic drugs”, 50.7% believed that “their lives would be impossible without biologic drugs”, 69.3% believed that “without biologic drugs, they would be very ill”, 61.3% believe that “their health in the future will depend on biologic drug”, and 85.3% believe that “their biologic drug protects them from becoming worse”.
- Concerning the “specific concern” item, its mean±SD (range) score was 10.3±3.3 [5.0–25.0]. Most patients (90.7%) didn’t believe that “having to take biologic drugs worries them”, 52.0% did’t believe that “they sometimes worry about long-term effects of their biologic drug”, 46.7% were uncertain about “their biologic drug is a mystery to them”, 88.0% didn’t believe that “their biologic drug disrupts their life”, and 60.0% didn’t believe that “they sometimes worry about becoming too dependent on their biologic drug”.

**Table 3.** Distribution of responses for each item on beliefs about medicines questionnaire for general and specific beliefs (n=75).

	**Agree/strongly agree (n=75)**	**Uncertain (n=75)**	**Disagree/strongly disagree (n=75)**
**General overuse**			
Doctors use too many medicines	62 (82.7)	2 (2.7)	11 (14.7)
Natural remedies are safer than medicines	3 (4.0)	3 (4)	69 (92.0)
Doctors place too much trust on medicines	66 (88.0)	7 (9.3)	2 (2.7)
If doctors had more time with patients, they would prescribe fewer medicines	44 (58.7)	13 (17.3)	18 (24.0)
**General harm**			
People who take medicines should stop their treatment for a while every now and again	10 (13.3)	3 (4)	62 (82.7)
Most medicines are addictive	54 (72.0)	9 (12)	12 (16.0)
Medicines do more harm than good	20 (26.7)	18 (24)	37 (49.3)
All medicines are poisons	19 (25.3)	20 (26.7)	36 (48.0)
**Specific necessity**			
My health, at present, depends on my biologic drug	58 (77.3)	10 (13.3)	7 (9.3)
My life would be impossible without my biologic drug	38 (50.7)	14 (18.7)	11 (14.7)
Without my biologic drug, I would be very ill	52 (69.3)	16 (21.3)	9 (12.0)
My health in the future will depend on my biologic drug	46 (61.3)	16 (21.3)	13 (17.3)
My biologic drug protects me from becoming worse	64 (85.3)	4 (5.3)	7 (9.3)
**Specific concern**			
Having to take biologic drugs worries me	3 (4.0)	4 (5.3)	68 (90.7)
I sometimes worry about long term effects of my biologic drug	18 (24.0)	18 (24)	39 (52.0)
My biologic drug is a mystery to me	23 (30.7)	35 (46.7)	17 (22.7)
My biologic drug disrupts my life	4 (5.3)	5 (6.7)	66 (88.0)
I sometimes worry about becoming too dependent on my biologic drug	12 (16.0)	18 (24)	45 (60.0)

Data in number (%).

**Table 4** details the impact of the disease characteristics on the patients’ specific beliefs about the biologic drug. Its main conclusions: i) disease characteristics have no impact on the specific concern score, and ii) only the marital status impacts the specific necessity score: compared to married patients, divorced or widowed ones had a lower score (19.8±3.2 vs. 18.6±4.4, respectively). **Table 5** details the correlation between the specific necessity score, patients reported outcome, disease activity, and functional impairment. Fair correlations were found with swollen joint count, tender joint count, VAS, disease activity score 28- CRP, the number of nocturnal awakenings, and patient global evaluation. The correlation with the HAQ was weak.

**Table 4.** Impact of disease characteristics on patients’ specific beliefs about the biologic drug (n=75).

**Parameter**	**Unit/category**	**Specific necessity score**	**p-value**	**Specific concerns score**	**p-value**
**Sex**	Female	19.4±3.3	0.874	9.2±5.2	0.874
	Male	20.5±3.1		9.9±7.3	
**Schooling level**	Illiteracy	20.4±2.8	0.570	10.3±5.2	0.540
	Primary school	19.1±3.7		8.9±6.3	
	Secondary school	19.3±3.4		8.3±4.1	
	University	19.6±2.3		11.6±11.5	
**Profession**	Unemployed	19.7±3.0	0.921	9.2±5.0	0.716
	Employee	19.3±4.0		9.3±6.7	
	Retired	20.7±2.0		10.5±9.7	
**Marital status**	Celibate	18.6±1.6	0.002# a	13.8±7.8	0.548
	Married	19.8±3.2		8.4±5.0	
	Divorced/widowed	18.6±4.4		15.6±5.3	
**Living area**	Urban	19.5±3.3	0.597	6±5.8	0.096
	Rural	20.1±3.1		7.0±4.0	
**Extra-articular manifestation**	Yes	20.25±2.9	0.567	9.7±5.9	0.595
	No	19.3±3.4		9.1±5.6	
**Coxitis**	Yes	17.0±2.0	0.265	10.3±2.9	0.635
	No	19.7±3.3		9.3±5.7	
**Atlanto-axial dislocation**	Yes	18.5±4.0	0.611	7.6±4.6	0.314
	No	19.7±3.2		9.5±5.7	
**Route administration of biologic modifying antirheumatic drugs**	Subcutaneous	20.0±3.7	0.128	8.6±5.9	0.763
	Intra-venous	19.2±2.7		10.2±5.3	
**Conventional synthetic modifying antirheumatic drugs**	Yes	19.7±3.5	0.209	9.4±4.9	0.244
	No	19.6±3.1		9.2±6.1	

Data were mean±standard deviation.

p-values (student test) <0.05: comparison between 2 groups.

# p-values (one-way analysis of variance) <0.05. Tukey’s post-hoc test < 0.05:

a married vs. divorced/widowed.

**Table 5.** Impact of patient reported outcome, disease activity, and functional impairment on the specific necessity score (n=75).

	**Pearson correlation coefficient (r)**	**p-value**
**Patient global evaluation**	0.307^a^	0.007
**Visual analogue scale**	0.367^a^	0.001
**Number of nocturnal awakenings**	0.319^a^	0.005
**Tender joint count**	0.380^a^	0.001
**Swollen joint count**	0.441^a^	0.001
**Health assessment questionnaire**	0.264^b^	0.022
**Disease activity score 28- C-reactive protein**	0.366^a^	0.001

“Pearson r”:

a “fair” if it was between 0.30–0.50; and

b “weak or no association” if it was < 0.30.

**Table 6** summarises the relationship between adherence and BMQ parameters. Compared to the adherent group, the nonadherent one had a lower specific necessity score and a higher specific concern score, and similar scores for general overuse and general harm. In logistic regression, specific necessity and specific concerns were significantly associated with adherence (OR= 0.855, 95% CI [0.726–1.006] and 1.438, 95% CI [1.004–1.980], respectively).

**Table 6.** Relationship between adherence and parameters of the “beliefs about medicines” questionnaire (n=75).

	**Adherent (n=71)**	**Non-adherent (n=4)**	**p-value**
**Specific necessity score**	21.0±5.4	13.5±7.5	0.009*
**Specific concerns score**	10.1±3.13	15.0±2.8	0.036*
**General overuse score**	10.0±2.3	9.5±1.9	0.600
**General harm score**	13.3±4.4	11.5±4.1	0.400

Data were means ± standard deviation

* p-value (student’s test): comparison between the 2 groups.

## DISCUSSION

The main conclusions of this study including 75 RA patients were: i) High adherence to biologic drugs (94.7%); ii) Compared to the adherent group (n=71), the non-adherent group (n=4) had a lower specific necessity score (21.0±5.4 vs. 13.5±7.5, respectively), a higher specific concerns score (10.1±3.13 vs. 15.0±2.8, respectively), and similar scores for general overuse and general harm; and iii) Specific necessity and specific concerns scores were significantly associated with adherence (OR= 0.855, 95%CI [0.726–1.006], and 1.438, 95%CI [1.004–1.980], respectively).

Assessment of patients' beliefs regarding treatment of chronic diseases is paramount because they can influence treatment adherence.^33^ However, in the literature, few studies have focused on determining therapeutic beliefs in RA patients, especially regarding biologic drugs. A systematic review conducted by Pasma et al. reported that psychosocial factors, such as beliefs about the necessity to take medication are strongly associated with therapeutic adherence in RA patients.^5^

### Patients’ adherence and beliefs

Adherence to bDMARDs are highly variable, ranging from 30 to 89%.^7–9^ Adherence to subcutaneous (S/C) bDMARDs and Oral Drugs for RAin the ARCO Study (Study on Adherence of Rheumatoid Arthritis patients to SubCutaneous and Oral Drugs), was 85.7%.^34^ It was reported that patients on biologics showed higher adherence than those on first-line csDMARDs (64.4% vs. 43.1%, respectively).^35^ In our study, adherence to the current biologic therapy was reported by 94.7% of patients. This high percentage can be explained using a self-reported questionnaire. Indeed, less adherent patients may record themselves as adherent. Adherence to different therapies among RA patients is influenced by many factors, including their sociodemographic conditions, the associated comorbidities and their treatments, rheumatic disease variables and activity, their individual perceptions about illness and about drugs, and the associated treatment for RA and their burden.^10^

In our study, no differences were observed between adherent and non-adherent patients regarding any of the sociodemographic factors, except for marital status. Previous studies have shown great variability in results and, sometimes, contradictory ones about the influence of these factors on treatment adherence.^5,36^ This variability can be partially explained by the regional characteristics, study design, or the different methods used to assess treatment adherence. However, family and professional support has been reported to be associated with an improvement in adherence to bDMARDs in RA, which is confirmed by our results.^37,38^ Regarding therapeutic modalities, there were no differences in adherence among patients receiving combination therapy and those receiving monotherapy, nor between patients on intravenous (IV) bDMARDs (IL-6Ri) and those on S/C bDMARDs. In their study, Markatseli et al. have reported that the main reasons for not adhering to IL-6Ri are non-medical, with drug supply being the most common issue.^37^

Beliefs and perceptions of RA patients may play a critical role in their adherence behavior.^39^ Specific beliefs about anti-rheumatic drugs are independent factors of adherence.^6,39–43^ In accordance with the literature, our study reported a strong association between specific necessity and specific concerns about bDMARD and adherence behavior.^39^ In fact, BMQ score factors (especially increased medication necessity), lower medication concerns, and increased treatment control, are independently predictive of increased adherence.^38^ In some studies, medication beliefs and illness perceptions predictive factors of adherence stronger than sociodemographic or clinical factors.^38,43^ Moreover, increased belief in the necessity to take medication was related to better treatment adherence.^29,44^ However, other authors, investigating patients' beliefs about S/C bDMARDs, found no difference between adherent and non-adherent patients regarding necessity or concern scores.^41^ Additionally, specific concerns, general overuse, and general harm scores were found to be higher in non compliant patients.^6,28^ In contrast, in our study, general beliefs did not influence adherence. Indeed, it is crucial to make some interventions. Effective patient education using different patient-specific modalities can help reduce the confusion associated with the disease and treatment modalities and improve patient adherence to their treatment.^45,46^ Indeed, in their study, Kumar et al. emphasised that patients reporting lower adherence were more dissatisfied with the information they had received about their DMARDs and had more negative beliefs about their treatment.^47^ More recently, studies reported that a good communication with health professionals, health professional support, and a better explanation of the risks of RA to patients’ health would all promote better treatment adherence.^48,49^

### Influencing factors on patients’ beliefs

Beliefs can be evaluated by specific tools such as the BMQ, which is a valid rheumatology questionnaire that can evaluate general and specific beliefs related to treatment.^29^ Some authors suggested that the BMQ can predict non-adherence.^40^ To the best of the authors’ knowledge, no previous studies reported these findings in RA patients under biologic drugs, or reported data regarding the “general harm” score.

In this study, most patients “agreed/strongly agreed” that doctors overuse the medicines and that they place too much trust on medicine. Most patients “agreed/strongly agreed” that medicines are addictive. Most of them “disagreed/strongly disagreed'' that medicines do more harm than good or that all medicines are poisons. Our results are in accordance with the literature.^27,35,50–52^ In the study conducted by Mucke et al. dealing with patients’ beliefs on DMARDs, the median of “general overuse” was about 12, and the median of “general harm” was about 10.^27^ There were no excessive negative views about doctors' attitudes and prescriptions. In the study conducted by Nestoriuc et al. “general harm” and “general overuse” scales were less than specific necessity and specific concerns.^51^ In the study conducted by van Heuckelum et al. the mean score for therapeutic necessity and concern related to DMARDs was 19.9 and 14.1, respectively, with a mean necessity-concern differential score of 5.8.^52^ This suggests that positive beliefs outweigh negative ones.^52^

In another study, patients with RA on DMARDs had higher necessity scales comparably to concern.^27^

Factors that may influence beliefs about biologic drugs among RA patients were poorly studied in the literature.

In the study conducted by Cea-Calvo et al. socio-demographic parameters and RA variables did not have significant influence on patients’ beliefs.^50^ Additionally, there were no important differences in beliefs according to the biologic drug characteristics such as the interval of administration and the rank of current biologic.^50^ In a similar vein, in the current study, there was no impact of sociodemographic characteristics and therapeutic modalities on patients’ specific beliefs (necessity and concerns) about biologic drugs. However, the specific necessity scale was correlated with marital status, patient reported outcomes, disease activity, functional impairment, and the specific necessity score.

## STUDY LIMITATIONS

This study has some limitations. First, adherence and belief were measured transversely at a given time. Adherence is a dynamic parameter and a cross-sectional evaluation may therefore not be reflective of its evolution.^53^ Second, self-reported adherence is a good tool, but some patients may underestimate their oversight and less adherent patients may report themselves as highly adherent.^54^ Third, the relatively small sample size could be the cause of the lack of association between some of the variables studied and therapeutic adherence.

## CONCLUSION

Our study reported that RA patients have strong beliefs about the need for biologic drugs. Patient-reported outcomes, disease activity, and functional impairment may affect patients' medical beliefs. However, beliefs about biologic drugs have been identified as key contributors to adherence. Therefore, beliefs must be dealt with at an early stage. Educational programs that focus on patients’ beliefs regarding the necessity of medication may result in better adherence and in slowing disease progression, especially during the early stages of the disease.

## Data Availability

The data and supportive information are available within the article.
